# Efficacy of acupuncture for melasma: A protocol of systematic review and meta-analysis

**DOI:** 10.1097/MD.0000000000028298

**Published:** 2021-12-17

**Authors:** Liheng Tang, Jin Xian, Ye Zhang, Changyun Zhang, Huijuan Yu, Qiwen Tan, Xin Zhang

**Affiliations:** a Shandong University of Traditional Chinese Medicine, Jinan, Shandong, China; b Affiliated Hospital of Shandong University of Traditional Chinese Medicine, Jinan, Shandong, China.

**Keywords:** acupuncture, melasma, protocol, randomized controlled trials, systematic review and meta-analysis

## Abstract

**Background:**

Melasma is a common acquired hyperpigmentation of the skin. The clinical manifestations are light brown or dark brown patches symmetrically distributed on the cheeks, forehead, and mandibular with different shades and unclear borders. The treatment of melasma is extremely challenging due to its easy relapse and hard to cure. As a classical method of traditional Chinese medicine, acupuncture has been used to treat melasma in clinical practice. However, there is no systematic review and meta-analysis of acupuncture on melasma. This study aims to assess the efficacy of acupuncture for melasma.

**Methods:**

We will perform a comprehensive retrieval in the following electronic databases: PubMed, EMBASE, the Cochrane Central Register of Controlled Trials, China National Knowledge Infrastructure (CNKI), Wan Fang Database, VIP database, Chinese Biomedical Literature Service System (SinoMed), Chinese Biomedicine (CBM) database, and TCM Literature Analysis from inception to July 1, 2021, to identify any eligible study. We include all randomized controlled trials (RCTs) without any limitation of blinding or publication language, and exclude cohort studies and case reports. The meta-analyses will be conducted by using the Stata16. We will use the Cochrane risk of bias tool for randomized trials to assess the risk of bias of included studies. The X^2^ and *I*^2^ tests will be utilized to assess the statistical heterogeneity of evidence. We will conduct a meta-analysis if the studies can be combined. Otherwise, a narrative synthesis will be performed. Article screening and data extraction will be independently evaluated by 2 reviewers trained in methodology according to the established selection criteria. Any disagreements between the 2 reviewers will be resolved by reaching a consensus with the other authors (the third reviewer).

**Results:**

This study will systematically evaluate the clinical efficacy of acupuncture for melasma.

**Conclusion:**

The conclusion of this study will provide evidence to ensure the efficacy of acupuncture for melasma, so as to provide an objective and scientific basis for clinical practice.

**Ethics and dissemination:**

No ethical approval will be required because the data used are not linked to individual patient. The results of this review will be published in a peer-reviewed journal.

**Registration number:**

INPLASY2021110097.

## Abstract 2

Supplemental Digital Content is available in the text

## 1 Introduction

Melasma is a commonly acquired hypermelanosis that affects sun-exposed areas of the skin, with frequent facial involvement.^[1]^ The clinical manifestations are light brown or dark brown patches symmetrically distributed on the cheeks, forehead, and mandible with different shades and unclear borders.^[2]^ Melasma affects especially women during menstruation,^[3]^ especially in thirties and forties Asian women.^[4]^ The incidence rate of Asian women of childbearing age is as high as 30%.^[2]^ There are 3 types of melasma, which are classified according to their distribution on the face, including central facial, cheekbone, and mandibular type.^[5,6]^ But other sites may also be involved, forming extra-facial melasma.^[7]^ Earlier studies classified melasma as epidermis, dermis, or mixed according to Wood's lamp examination, but studies using laser confocal microscopy showed that all melasma are mixed, indicating that there is a common pathophysiology.^[1,8]^ Factors affecting melasma may include ultraviolet radiation, genetic factors, hormones, inflammation, reactive oxygen species,^[9]^ melanin synthesis increase, hyperplasia of blood vessels in skin lesions, skin barrier impairment, and basement membrane disruption.^[1]^ In addition, sleep disorder, using inferior cosmetics such as mercury and excessive lead content, exposing to heat radiation such as cooking, thyroid diseases, female reproductive system diseases, and liver diseases can also induce or aggravate melasma.^[2]^ Some studies have also reported melasma in patients following stressful events and affective disorder such as depression.^[10,11]^ Current treatments for melasma include topical medications, chemical strippers, laser and phototherapy, and systemic medications,^[12]^ options target photoprotection, melanocytes activity, various dermal or epidermal cells that signal to melanocytes, and abnormal tissue changes due to photoaging.^[13]^ Melasma is no longer considered to be a static process, but a complex epidermal–dermal dynamic interaction with various cell types, inflammation, oxidative stress, and photodamage all contribute significantly to this process.^[9]^ Despite a strong demand for treatment, the treatment of melasma remains highly challenging with inconsistent results and almost constant relapses.^[14]^ Chloasma belongs to the category of Chinese medicine “Soot black plaque,” “butterfly plaque,” “liver plaque,” “face dust,” and other diseases.^[15]^ Acupuncture has been used in clinical treatment of melasma, which may be related to acupuncture to dredge meridians, promote blood circulation and remove stasis, and promote circulation of qi and blood.^[16–18]^ Traditional Chinese medicine believes that the cause of this disease mainly lies in kidney-yang deficiency, liver stagnation and qi stagnation, spleen deficiency, etc. Acupuncture has a better therapeutic effect for this disease with fewer side effects, and is easy to be accepted by patients.^[19,20]^ According to published articles, there is a lack of high-quality evidence for the treatment of melasma with acupuncture, and no systematic review and meta-analysis have been conducted in this regard. Therefore, we will systematically evaluate the clinical efficacy of acupuncture for melasma, so as to provide an objective and scientific basis for clinical practice.

## 2 Methods

This systematic review protocol has been registered on INPLASY (registration number: INPLASY2021110097), and is available in full on the inplasy.com (https://doi.org/10.37766/inplasy2021.11.0097). This protocol has been checked with Preferred Reporting Items for Systematic review and Meta-Analysis Protocols (PRISMA-P) checklist.^[21]^ If the protocol is modified, we will describe the information in the final report.

### 2.1 Inclusion criteria

#### 2.1.1 Types of studies

We will include all randomized controlled trials (RCTs) of acupuncture for the treatment of melasma without any blinding or publication language restrictions. We will also exclude cohort studies, case reports, and duplicate publications.

#### 2.1.2 Types of participants

Participants will include patients diagnosed with melasma based on medical history and typical clinical manifestations: there are no restrictions on the age, gender, or race of the subject.

### 2.2 Types of interventions

#### 2.2.1 Experimental interventions

We will include all RCTs in which the treatment group uses acupuncture alone. Acupuncture treatment is defined as acupuncture at acupoints on the meridian, including manual acupuncture or electroacupuncture, but not other acupuncture therapies, such as ear acupuncture, scalp acupuncture, dry acupuncture, and acupressure therapy.

#### 2.2.2 Comparator interventions

The control group uses only conventional treatment. Conventional treatments include topical medications, chemical stripping agents, laser and light therapy, systemic medications, and other necessary treatments.

### 2.3 Types of outcome measures

#### 2.3.1 Primary outcome

The main outcomes include Melasma Area and Severity Index (MASI),^[22]^ quantification is carried out according to the area, color depth, and color uniformity of melasma. Pigmentation area assessment: forehead (F), right cheek (MR), left cheek (ML) and lower jaw (C) are divided into 4 areas, with weights of 30%, 30%, 30%, and 10%, respectively. The color depth (D) and uniformity (H) scores are counted as 0 to 4 points: 0 means nothing, 1 means slight, 2 means moderate, 3 means obvious, and 4 means maximum. MASI = forehead [0.3A (D + H)] + right cheek [0.3A (D + H)] + left cheek [0.3A (D + H)] + lower jaw [0.1A (D + H)]. The maximum is 48 points, the minimum is 0.

#### 2.3.2 Secondary outcome

Secondary results include scanning reflectance spectrophotometer detection technology (colorimetric method),^[3]^ VISIA image analysis, noninvasive physiological function test, reflectance confocal microscopy (RCM),^[23]^ dermoscopic observation and evaluation of the improvement of the number and shape of blood vessels in the skin lesions before and after treatment of melasma,^[24]^ Physician's Global Assessment (PGA),^[25]^ patient satisfaction evaluation, safety indicators, and the number of adverse events.

### 2.4 Search strategy

#### 2.4.1 Electronic searches

We will search PubMed, EMBASE, the Cochrane Central Register of Controlled Trials, China National Knowledge Infrastructure (CNKI), Wan Fang Database, VIP database, Chinese Biomedical Literature Service System (SinoMed), Chinese Biomedicine (CBM) database and TCM Literature Analysis and Retrieval Database from inception to July 1, 2021, to identify any eligible study. We include all RCTs without any limitation of blinding or publication language, exclude cohort studies and case reports. Detailed search strategy will be shown in Appendix 1, http://links.lww.com/MD2/A765.

#### 2.4.2 Searching other resources

We will search the US National Institutes of Health Ongoing Trials Register, the WHO International Clinical Trials Registry Platform, Chinese Clinical Trial Registry (ChiCTR), ClinicalTrials.gov, Google Scholar, and Baidu scholar for any relevant ongoing or unpublished trials.

### 2.5 Data collection and analysis

#### 2.5.1 Selection of studies

**Figure 1:**
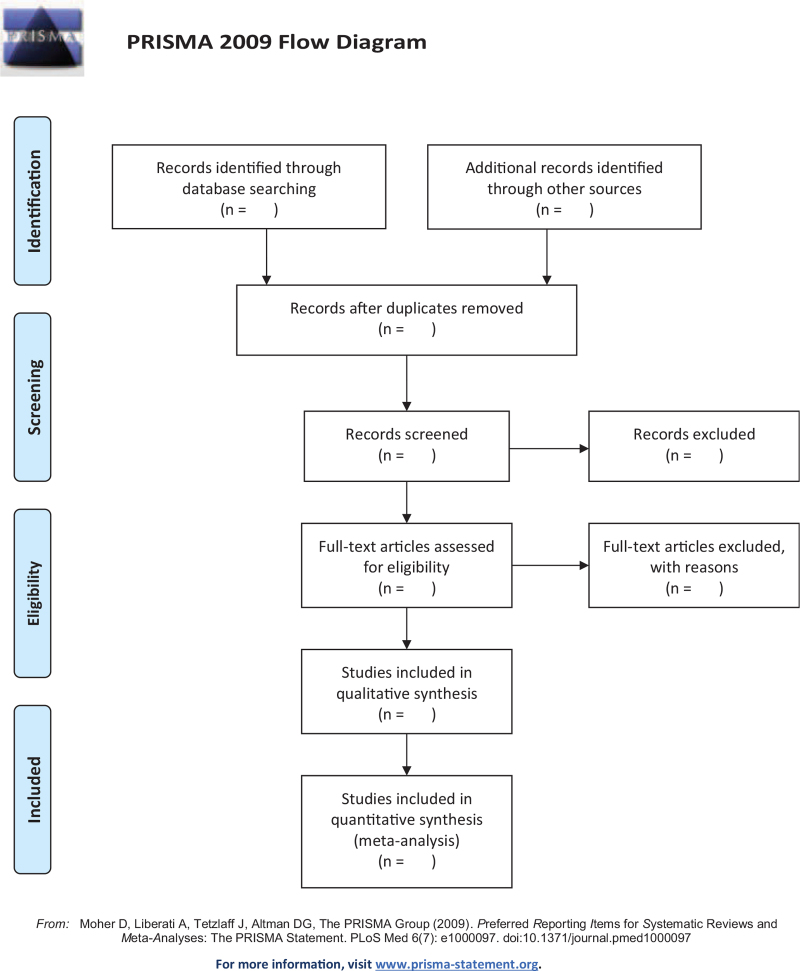
PRISMA flow diagram.

The titles and abstracts of all searched studies will be independently evaluated by 2 reviewers (CZ and HY) trained in methodology according to the established selection criteria. Any disagreements between the 2 reviewers will be resolved by reaching a consensus with the third reviewer (QT). If necessary, they will read the full text of all included studies. A PRISMA flowchart will be drawn to illustrate the study selection process (Fig. 1).

#### 2.5.2 Data extraction and management

Citations will be independently screened by the 2 authors (CZ and HY), and data will be extracted using a standardized data extraction table. The following information will be determined for each trial: authors name, publication year, inclusion and exclusion criteria, number of patients and reviews, type of acupuncture, and outcome measures. Any disagreement will be resolved by consensus or consultation with a third review author.

#### 2.5.3 Assessment of risk of bias in included studies

Two reviewers (CZ and HY) will independently assess the risk of bias using the Cochrane Risk of Bias Tool of Randomized Trials. They will compare their own assessments and discuss any differences of opinion between them. If they fail to do so, they will be arbitrated by a third-party reviewer (QT). Area to be evaluated: Is there enough sequence generation (selection bias)? Is the allocation sufficiently masked (selection bias)? During the course of the research, is there sufficient knowledge about the interventions allocated? Participants and personnel (performance bias), result assessor (detection bias); are incomplete result data adequately resolved (attrition bias)? Is there no hint of selective result reporting (reporting bias) in the research report? Is this study apparently free of other issues that might put it at risk of bias? On the basis of the relevant information extracted from each qualified study, the risk of bias in each area will be divided into high risk, low risk, and unclear risk of bias.

#### 2.5.4 Measures of treatment effect

We will conduct a meta-analysis if the studies can be combined. For dichotomous variables, a risk ratio (RR) with 95% confidence interval (95% CI) will be used for analysis. Standardized mean difference (MD) with 95% CI will be calculated for continuous data.

#### 2.5.5 Dealing with missing data

We will attempt to contact the original researchers via email to obtain any missing or inadequate data. Then, we will perform a sensitivity analysis using imputations of missing outcome data of dichotomous outcomes in best-worse and worse-best case scenarios to assess the potential impact of loss to follow-up. If we cannot collect accurate data, we will exclude these studies.

#### 2.5.6 Assessment of heterogeneity

The X^2^ and *I*^2^ tests will be utilized to assess the statistical heterogeneity of evidence.^[26]^ When *P* ≥ .1 and *I*^2^ ≤ 50%, it is considered that there is no statistical heterogeneity or the heterogeneity is small. When *P* < .1 and *I*^2^ > 50%, the result indicates that there is a statistical heterogeneity. We will calculate the *I*^2^ statistic (*I*^2^ values of 0–40% being interpreted as “might not be important”; 30–60%: may represent moderate heterogeneity; 50–90%: may represent substantial heterogeneity; and 75–100%: represents considerable heterogeneity). We will use subgroup analysis to explore the causes of heterogeneity among the results of studies.

#### 2.5.7 Assessment of publication biases

If more than 10 articles are included, we will use funnel plot and Egger test to assess publication bias. If there is publication bias, we will use the clipping-compensation method to further evaluate the impact of publication bias on the results. If the impact is not large, the authenticity of the results is better, and if the impact is relatively large (becomes non-statistically significant), it will be fully included in the results to discuss the impact of publication bias on the results.

#### 2.5.8 Data synthesis

If there is no heterogeneity, use a fixed-effects model to synthesize the data; if there is significant heterogeneity, use a random-effects model for analysis. We will provide a narrative synthesis of the outcomes and results of the studies if a meta- analysis is not possible.

#### 2.5.9 Subgroup analysis and investigation of heterogeneity

If a sufficient number of RCTs are included in the review, we plan to conduct subgroup analysis to explore the source of heterogeneity. The subgroup analysis will be based on the type of acupuncture (manual acupuncture or electroacupuncture) and the test time of the secondary results (3 or 7 days after the intervention).

#### 2.5.10 Sensitivity analysis

We plan to use the “leave-one-out” methods to conduct sensitivity analyses for the main outcomes to confirm the reliability of our findings.

#### 2.5.11 Quality of the evidence

The Grading of Recommendations Assessment, Development, and Evaluation (GRADE) method will be used to summarize the quality of evidence and provide a “Summary of Survey Results” table.^[27]^ The GRADE approach evaluates the quality of evidence as “high,” “moderate,” “low,” or “very low” by the outcome. The quality of evidence can be reduced by 5 factors (risk of bias, the inconsistency of results, indirectness of evidence, imprecision, and publication bias) and increase by 3 factors (large effect, dose response, opposing plausible residual bias, and confounding). The author will independently reduce or improve the quality of evidence and resolved disagreements by discussion.

#### 2.5.12 Amendments

We will provide the date of any amendment, a description of the change and the rationale in the event of protocol amendments.

#### 2.5.13 Patient and public involvement

Patient and public were not involved in this study.

#### 2.5.14 Ethics and dissemination

No ethical approval will be required because the data used are not linked to individual patient. The results of this review will be published in a peer-reviewed journal.

## 3 Discussion

Treatment of melasma is challenging, usually difficult to treat, and often recurs.^[6]^ Melasma has a significant impact on appearance, causing psychosocial and emotional distress, and reducing the quality of life of the affected patients. Patients commonly report feelings of shame, low self-esteem, listlessness, dissatisfaction, and the lack of motivation to go out, Suicidal ideas have also been reported in the literature.^[3]^ The mechanism of acupuncture treatment of melasma is still in its infancy and needs to be further developed. Preliminary studies have found that balancing oxidation and antioxidant functions, improving blood rheology, and regulating sex hormones may be an important mechanism of acupuncture treatment of melasma.^[28]^ We hope to evaluate the effectiveness of acupuncture in the treatment of melasma from published RCTs. The results of this review will help clinicians use acupuncture in the treatment of melasma.

## Author contributions

**Conceptualization:** Liheng Tang.

**Data curation:** Changyun Zhang, Huijuan Yu, Qiwen Tan.

**Formal analysis:** Changyun Zhang, Huijuan Yu, Qiwen Tan.

**Methodology:** Jin Xian.

**Project administration:** Xin Zhang.

**Resources:** Jin Xian, Ye Zhang.

**Software:** Liheng Tang, Jin Xian.

**Supervision:** Qiwen Tan, Xin Zhang.

**Visualization:** Liheng Tang, Jin Xian, Ye Zhang.

**Writing – original draft:** Liheng Tang.

**Writing – review & editing:** Ye Zhang, Xin Zhang.

## References

[1] Kwon SH Hwang YJ Lee SK. Heterogeneous pathology of melasma and its clinical implications. Int J Mol Sci 2016;17:824. 27240341 10.3390/ijms17060824 PMC4926358

[2] Expert consensus on diagnosis and treatment of melasma in China (2021 edition). Chin J Dermatol 2021;54:110–5.

[3] Handel AC Miot LD Miot HA. Melasma: a clinical and epidemiological review. An Bras Dermatol 2014;89:771–82. 25184917 10.1590/abd1806-4841.20143063 PMC4155956

[4] Kang HY Bahadoran P Suzuki I. In vivo reflectance confocal microscopy detects pigmentary changes in melasma at a cellular level resolution. Exp Dermatol 2010;19:e228–33. 20497220 10.1111/j.1600-0625.2009.01057.x

[5] Pagan RM Sanchez JL. Mandibular melasma. P R Health Sci J 2000;19:231–4. 11076368

[6] Sanchez NP Pathak MA Sato S. Melasma: a clinical, light microscopic, ultrastructural, and immunofluorescence study. J Am Acad Dermatol 1981;4:698–710. 6787100 10.1016/s0190-9622(81)70071-9

[7] Miot LD Miot HA Silva MG. Physiopathology of melasma. An Bras Dermatol 2009;84:623–35. 20191174 10.1590/s0365-05962009000600008

[8] Newcomer VD Lindberg MC Sternberg TH. A melanosis of the face (“chloasma”). Arch Dermatol 1961;83:284–99. 13728642 10.1001/archderm.1961.01580080114013

[9] Sarkar R Bansal A Ailawadi P. Future therapies in melasma: what lies ahead? Indian J Dermatol Venereol Leprol 2020;86:08–17. 10.4103/ijdvl.IJDVL_633_18 31793496

[10] Tamega AD Miot LD Bonfietti C. Clinical patterns and epidemiological characteristics of facial melasma in Brazilian women. J Eur Acad Dermatol Venereol 2013;27:151–6. 22212073 10.1111/j.1468-3083.2011.04430.x

[11] Wolf R Wolf D Tamir A. Melasma: a mask of stress. Br J Dermatol 1991;125:192–3. 1911305 10.1111/j.1365-2133.1991.tb06075.x

[12] Sheth VM Pandya AG. Melasma: a comprehensive update: Part II. J Am Acad Dermatol 2011;65:699–714. 21920242 10.1016/j.jaad.2011.06.001

[13] Kwon SH Na JI Choi JY. Melasma: updates and perspectives. Exp Dermatol 2019;28:704–8. 30422338 10.1111/exd.13844

[14] Passeron T Picardo M. Melasma, a photoaging disorder. Pigment Cell Melanoma Res 2018;31:461–5. 29285880 10.1111/pcmr.12684

[15] Lang WY Li YP Yang F. Status quo of acupuncture in the treatment of melasma. Gansu Sci Technol 2019;35:145–8.

[16] Liu HH Ni GX. Clinical study of through adjust sanjiao acupuncture combined with peri-acupuncture in the treatment of female melasma. Clin J Acupunct Moxib 2018;34:08–11.

[17] Dong Y. Clinical research progress of acupuncture and cosmetology. Jilin Trad Chin Med 2009;29:457–8.

[18] Yang XQ Wang X. Research progress of acupuncture cosmetology. Jilin Trad Chin Med 2017;37:855–7.

[19] Wu PP Zhao LH Chen WL. Acupoint selection rule of acupuncture and moxibustion in the treatment of melasma. Chin Med Guide 2019;25:94–8.

[20] Wu X Xiang Y. The effects of acupuncture combined with auricular acupressure in the treatment of chloasma. Evid Based Complement Alternat Med 2018;2018:6438458. 29849716 10.1155/2018/6438458 PMC5937619

[21] Shamseer L Moher D Clarke M. Preferred reporting items for systematic review and meta-analysis protocols (PRISMA-P) 2015: elaboration and explanation. BMJ 2015;350:g7647. 25555855 10.1136/bmj.g7647

[22] Pandya AG Hynan LS Bhore R. Reliability assessment and validation of the Melasma Area and Severity Index(MASI) and a new modified MASI scoring method. J Am Acad Dermatol 2011;64:78–83. 83.e1-2. 20398960 10.1016/j.jaad.2009.10.051

[23] Kang HY Bahadoran P. Application of in vivo reflectance confocal microscopy in melasma classification. J Am Acad Dermatol 2012;67:157 author reply 157-158. 22703910 10.1016/j.jaad.2012.02.046

[24] Khunger N Kandhari R Singh A. A clinical, dermoscopic, histopathological and immunohistochemical study of melasma and facial pigmentary demarcation lines in the skin of color. Dermatol Ther 2020;33:e14515. 33169501 10.1111/dth.14515

[25] Cestari T Arellano I Hexsel D. Melasma in Latin America: options for therapy and treatment algorithm. J Eur Acad Dermatol Venereol 2009;23:760–72. 19646135 10.1111/j.1468-3083.2009.03251.x

[26] Higgins JP Thompson SG. Quantifying heterogeneity in a meta-analysis. Stat Med 2002;21:1539–58. 12111919 10.1002/sim.1186

[27] Schünemann HJ, Higgins JP, Vist GE, *et al*. Chapter 14: Completing ‘Summary of findings’ tables and grading the certainty of the evidence. Draft version (29 January 2019) for inclusion. In: Higgins JP, Thomas J, Chandler J, et al (editors). Cochrane Handbook for Systematic Reviews of Interventions. London: Cochrane.

[28] Fu SS Qi CJ Shen L. Present situation and prospect of acupuncture treatment of melasma. Clin Study Chin Med 2019;11:145–8.

